# Association of Social Media Recruitment and Depression Among Racially and Ethnically Diverse Metabolic and Bariatric Surgery Candidates: Prospective Cohort Study

**DOI:** 10.2196/58916

**Published:** 2025-04-10

**Authors:** Jackson M Francis, Sitapriya S Neti, Dhatri Polavarapu, Folefac Atem, Luyu Xie, Olivia Kapera, Matthew S Mathew, Elisa Marroquin, Carrie McAdams, Jeffrey Schellinger, Sophia Ngenge, Sachin Kukreja, Benjamin E Schneider, Jaime P Almandoz, Sarah E Messiah

**Affiliations:** 1 Peter O’Donnell Jr. School of Public Health, University of Texas Southwestern Medical Center, 5323 Harry Hines Blvd, Dallas, TX, 75390-9066, United States, 1 9725462920; 2 School of Public Health, University of Texas Health Science Center at Houston (UTHealth), Dallas, TX, United States; 3 Department of Biostatistics & Data Science, School of Public Health, University of Texas Health Science Center at Houston, Dallas, TX, United States; 4 School of Public Health, The University of Texas at Austin, Austin, TX, United States; 5 Department of Nutritional Sciences, Texas Christian University, Fort Worth, TX, United States; 6 Department of Psychiatry, University of Texas Southwestern Medical Center, Dallas, TX, United States; 7 Division of Endocrinology, Department of Internal Medicine, University of Texas Southwestern Medical Center, Dallas, TX, United States; 8 Department of Surgery, University of Texas Southwestern Medical Center, Dallas, TX, United States; 9 Department of Pediatrics, University of Texas Southwestern Medical Center, Dallas, TX, United States

**Keywords:** social media, social media recruitment, depression, depressed, major depressive disorder, MDD, depressive disorder, depressive, race, racial, racial differences, ethnic, ethnic differences, ethnicity, metabolic surgery, bariatric surgery, obesity, obese, online health, ethics, mental health, eHealth, internet, digital health, digital mental health, online interventions, public engagement, public health

## Abstract

**Background:**

Due to the widespread use of social media and the internet in today’s connected world, obesity and depression rates are increasing concurrently on a global scale. This study investigated the complex dynamics involving social media recruitment for scientific research, race, ethnicity, and depression among metabolic and bariatric surgery (MBS) candidates.

**Objective:**

This study aimed to determine (1) the association between social media recruitment and depression among MBS candidates and (2) racial and ethnic differences in social media recruitment engagement.

**Methods:**

The analysis included data from 380 adult MBS candidates enrolled in a prospective cohort study from July 2019 to December 2022. Race and ethnicity, recruitment method (social media: yes or no), and depression status were evaluated using *χ*^2^ tests and logistic regression models. Age, sex, and ethnicity were adjusted in multivariable logistic regression models.

**Results:**

The mean age of the candidates was 47.35 (SD 11.6) years, ranging from 18 to 78 years. Participants recruited through social media (n=41, 38.32%) were more likely to report past or current episodes of depression compared to nonsocial media–recruited participants (n=74, 27.11%; *P=*.03), with a 67% increased likelihood of depression (odds ratio [OR] 1.67, 95% CI 1.04‐2.68, *P*=.03). Further analysis showed that participants with a history of depression who were below the mean sample age were 2.26 times more likely to be recruited via social media (adjusted OR [aOR] 2.26, 95% CI 1.03‐4.95; *P*=.04) compared to those above the mean age. Hispanic (n=26, 38.81%) and non-Hispanic White (n=53, 35.10%) participants were significantly more likely to be recruited via social media than non-Hispanic Black (n=27, 18.37%) participants (*P*<.001). After adjusting for covariates, non-Hispanic Black participants were 60% less likely than non-Hispanic White participants to be recruited via social media (aOR 0.40, 95% CI 0.22‐0.71; *P=*.002).

**Conclusions:**

We found that individuals recruited through social media channels, especially younger participants, were more likely to report past or current episodes of depression compared to those recruited through nonsocial media. The study also showed that non-Hispanic Black individuals are less likely to engage in social media recruitment for scientific research versus other racial and ethnic groups. Future mental health–related studies should consider strategies to mitigate potential biases introduced by recruitment methods to ensure the validity and generalizability of research findings.

## Introduction

Over the past 2 decades, obesity (ie, BMI ≥30 kg/m^2^) prevalence among adults in the United States has risen from 30.5% to 42.4% [1], and has nearly tripled worldwide [2]. Further, the prevalence of severe obesity (BMI ≥40 kg/m^2^) has doubled from 4.7% to 9.2% [3]. Non-Hispanic Black (NHB) adults are disproportionately impacted, with an age-adjusted obesity prevalence of 49.9%, followed by Hispanic (45.6%), non-Hispanic White (NHW) (41.4%), and non-Hispanic Asian (16.1%) individuals [3].

Various obesity treatment options, including diet control, exercise, cognitive and family-based behavioral therapy, pharmacological therapy, and metabolic and bariatric surgery (MBS) have been explored to address this critical issue. Of these, MBS is a safe and effective treatment option, particularly for individuals with severe obesity who are resistant to other interventions [4]. A 2014 meta-analysis of 164 studies involving 160,000 patients found that BMI decreased by at least 11.8 kg/m² one-year post MBS, with sustained weight loss observed five years post surgery [5]. Most individuals who undergo MBS experience a reduction or complete elimination of obesity-related health issues, including high blood pressure, heart disease, diabetes, asthma, and osteoarthritis [6,7]. Additionally, long-term outcomes of MBS in adolescents aged 16‐21 years demonstrated sustained weight reduction and improvement in comorbidities, including reduced depression prevalence over a decade post surgery [7]. Given the increasing prevalence of obesity and the proven safety, effectiveness, and long-term success of MBS, there has been an increase in the number of MBS procedures being performed, resulting in a parallel increase in MBS-related research [8,9]. However, the COVID-19 pandemic led to a temporary decrease in MBS surgeries due to delays in elective procedures [10]. Research studies conducted during the pandemic faced unique challenges, prompting a shift toward innovative recruitment methods. Researchers have increasingly used social media for participant recruitment, survey administration, and trend analysis. Although literature reviews underscore the efficacy of social media as a recruitment methodology, its use comes with challenges and drawbacks [11,12].

This study acknowledges the dual nature of social media, which, while a potent recruitment tool, has also been implicated in mental health and body image concerns. Celebrities and influencers on social media platforms have contributed to rising negative body image perceptions [13], which have been associated with higher depressive symptoms, loss of confidence, loneliness, and risk of developing eating disorders [13,14]. Studies have shown that viewing images that have been heavily edited can negatively impact an individual’s body image and that editing selfies—regardless of whether they are posted—was linked to self-objectification and dissatisfaction with facial features [15]. Additionally, overall time spent on image-based social media platforms has been associated with weight-bias internalization [16]. A meta-analysis examining the relationship between body image and eating disorders found that both body checking and body image avoidance were associated with eating disorder pathology [17].

Acknowledging the gaps in our understanding of social media’s impact on mental health among individuals with severe obesity and who are considering MBS, this study aimed to determine (1) the association between social media recruitment and depression among MBS candidates and (2) racial and ethnic differences in social media recruitment engagement. It was hypothesized that depression prevalence would be higher in individuals recruited through social media and that no significant racial and ethnic differences would exist between social media– and nonsocial–based media recruitment.

## Methods

### Study Design and Eligibility Criteria

The Bariatric Health Study (NIH/NIMHD 5R01MD011686) is an ongoing prospective cohort research study enrolling participants who qualify and have been referred for MBS based on established medical criteria. The American Society for Metabolic and Bariatric Surgery recommends MBS for individuals with a BMI >35 kg/m^2^, regardless of the presence or absence of comorbidities; for individuals with type 2 diabetes and a BMI >30 kg/m^2^; or for individuals with a BMI between 30‐34.9 kg/m^2^ who have not achieved adequate weight loss or improvement in comorbidities with nonsurgical obesity treatment methods [18]. Eligibility criteria mandated that participants be at least 18 years old, medically eligible for MBS, and referred for surgery by a clinical provider. To manage and collect study data securely and in compliance with HIPAA (Health Insurance Portability and Accountability Act of 1996) regulations, we used REDCap (Research Electronic Data Capture; Vanderbilt University), a web-based database management platform [19,20].

### Ethical Considerations

The study was approved by the University of Texas Health System Institutional Review Board (IRB# HSC-SPH-18‐0850) and the Committee for the Protection of Human Subjects. Informed consent was obtained from all study participants. Participant data are stored in a HIPAA-compliant, password-protected, encrypted database with limited access. The data used for this analysis were deidentified. Study participants were compensated with a US $50 gift card upon completion of the initial Mini International Neuropsychiatric Interview (MINI) and surveys. Upon surgery completion, participants were compensated at 6, 12, and 24 months with US $100, US $125, and US $150 gift cards for completing the additional MINI interviews and surveys.

### Participant Recruitment

Recruitment for this study was open from August 2019 to October 2022. Recruitment sites for this prospective cohort study consisted of MBS specialty clinics in the North Texas region. These included Minimally Invasive Surgical Associates, Lee Bariatrics Clinic, City Hospital at White Rock, Dallas, TX, and the UT Southwestern Weight Wellness Subspecialties Clinic, Dallas, TX. Before the COVID-19 pandemic, eligible MBS patients were recruited by research coordinators during mandated educational seminars held by MBS team members before surgery. Research coordinators provided interested candidates with information and a sign-up sheet at these seminars. In addition, the institutional review board–approved brochures and flyers were placed in the MBS clinic waiting rooms. Interested participants completed a web-based eligibility screener and were contacted by research coordinators to complete informed consent and baseline measurements. However, these educational seminars transitioned to virtual platforms due to the pandemic. Additionally, social media channels were added as a recruitment source to meet our recruitment targets, as elective surgeries at our academic center were paused on several occasions due to surges in COVID-19. Specifically, web-based social media recruitment was conducted through Facebook and Instagram and was managed by the study’s research coordinators. Targeted advertisements on these platforms directed outreach toward individuals meeting our inclusion criteria. Interested individuals completed the study’s online eligibility screener to ensure that the inclusion criteria were met. Following this, informed consent was completed, and research coordinators performed baseline measurements. To sustain study engagement, biweekly informative videos and posts addressing topics related to MBS were created.

### Main Outcome

The primary outcome measure was self-reported depression, measured by the MINI [21], a validated tool for evaluating major psychiatric disorders, comprising dichotomous questions with follow-up questions for positive responses [21]. Previous studies have demonstrated that the MINI accurately diagnoses psychiatric conditions [22]. The interview primarily features “yes” or “no” questions, with additional clarifications provided to participants when necessary. Following the completion of questions for each psychiatric disorder, research coordinators (interviewers) tallied the “yes” and “no” responses to identify if the interviewee screened positively for a variety of potential psychiatric disorders. In this study, we assessed current, past, and recurrent major depressive episodes, posttraumatic stress disorder, agoraphobia, social anxiety disorder, general anxiety disorder, and panic disorder. Following the initial interview, research coordinators determined the presence or absence of depression based on participants’ responses. Participants who screened positive for a current, past, or recurrent major depressive episode during the MINI interview were classified as having a history of depression. Conversely, if none of these conditions were selected (current, past, or recurrent), participants were categorized as not having a history of depression.

### Main Exposure

We assessed each participant’s recruitment method through the enrollment survey. Participants responded to the question, “How did you learn about this study?” They could choose from options such as “Lee Bariatrics, Texas Presbyterian Hospital, UT Southwestern Medical Center, Minimally Invasive Surgical Associates, UTSW Weight Wellness Program, City Hospital at White Rock, Flyer, Brochure or Pamphlet, Social media (ie, Facebook, Instagram), or Other.” We classified participants as positive for social media recruitment if they chose “Social media (ie, Facebook, Instagram)” or mentioned social media recruitment in the “Other” section. We categorized participants as negative for social media recruitment if they selected other options.

### Covariates

Covariates including age, sex, race and ethnicity, BMI, education level, and perceived financial well-being were obtained through self-report. Age was treated as a continuous variable, representing the participants’ age in years. Sex was a categorical variable, categorized as male, female, or other. Race and ethnicity were also categorical variables, encompassing diverse categories such as NHW, NHB, Hispanic, and Other. BMI was calculated via self-reported height and weight and verified via medical records provided by participants when available. Education was a categorical variable, categorized as none, elementary school, high school, college, and graduate or professional degree. In our survey, we did not have a direct measure of income. However, as a proxy measure, we asked the question, “In the last two weeks, have you had enough money to meet your needs?” This variable was categorical, with answers being “not at all, a little, moderately, mostly, and completely.” These covariates served as essential demographic factors for our analysis, providing a comprehensive understanding of their potential influence on the investigated variables.

### Statistical Analysis

We analyzed descriptive variables, including sex, age, race and ethnicity (ie, NHB, NHW, Hispanic), BMI, and history of depression, comprising both categorical and continuous variables. To explore the associations between recruitment methods, social media or nonsocial media, and depression history (yes/no), we conducted Pearson *χ*^2^ tests. Crude odds ratios were calculated using univariable logistic regression models to assess the relationship between recruitment method and depression history. Furthermore, we performed multivariable logistic regression by adjusting for age, race and ethnicity, and sex to calculate the adjusted odds ratios for depression in relation to the recruitment method used. There were less than 3% missing data for demographic variables (ie, age, sex, and ethnicity); no data was missing for the outcome variable. We performed all analyses using STATA (v.17.1, Stata Corp LP), with statistical significance set at a *P* value below 5%.

## Results

### Participant Characteristics

Participant characteristics (N=380) stratified by recruitment method, social media (n=107), and nonsocial media (n=273) are presented in Table 1. Participants recruited through social media had a mean age of 47.27 (SD 1.02) years, whereas those in the nonsocial media group had a mean age of 47.38 (SD 0.73) years (*P*=.93). Sex distribution in the social media group was 9 (8.41%) males and 98 (91.59%) females, while the nonsocial media group included 41 (15.02%) males and 232 (84.98%) females, *P*=.09. The mean BMI for the social media group was 44.92 (SD 1.05) kg/m^2^ and the nonsocial media group was 45.68 (SD 0.64) kg/m^2^ (*P*=.54). Regarding racial and ethnic backgrounds, the social media group had 49.53% (n=53) of participants identifying as NHW, 25.23% (n=27) as NHB, 24.3% (n=26) as Hispanic, and 0.93% (n=1) as other. In the nonsocial media group, 35.9% (n=98) were NHW, 43.96% (n=120) were NHB, 15.02% (n=41) were Hispanic, and 5.13% identified as Other (*P*<.001).

**Table 1.** Baseline characteristics of metabolic and bariatric surgery candidates recruited via social media and traditional methods in a prospective cohort study (N=380, 2019‐2022).

Variables	Participants (N=380)		*P* value
	Social media (n=107)	Nonsocial media (n=273)	
Age (years), mean (SD)	47.27 (1.02)	47.38 (0.73)	.93
Sex, n (%)			.09
Males (n=50, 13.16%)	9 (8.41)	41 (15.02)	
Female (n=330, 86.84%)	98 (91.59)	232 (84.98)	
BMI (kg/m^2^), mean (SD)	44.92 (1.04)	45.68 (0.64)	.54
Race/ethnicity, n (%)			<.001^a^
Non-Hispanic White (n=151)	53 (49.53)	98 (35.9)	
Non-Hispanic Black (n=147)	27 (25.23)	120 (43.96)	
Hispanic (n=67)	26 (24.3)	41 (15.02)	
Other (n=15)	1 (0.93)	14 (5.13)	

a Significant.

### Depression Prevalence by Recruitment Method

Table 2 presents the prevalence of depression categorized by recruitment method and race and ethnicity among all participants (N=380). Of the participants, 115 (30.26%) had a history of depression, while 265 (69.74%) had no history of depression. Our findings showed a difference between the prevalence of depression and the recruitment methods (*P*=.03).

In the nonsocial media group (n=273, 71.84%), the prevalence rates of depression (n=74, 27.11%) varied by race and ethnicity. In the NHW subgroup (n=98), 26 (26.53%) participants had a history of depression, while 72 (73.47%) did not. In the NHB subgroup (n=120), 28 (23.33%) participants had a history of depression, whereas 92 (76.67%) had no history. Among the Hispanic subgroup (n=41), 15 (36.59%) participants had a history of depression, while 26 (63.41%) had no history. In the “Other” subgroup (n=14), 5 (35.71%) participants had a history of depression, while 9 (64.29%) had no history.

Within the social media group (n=107, 28.16%), subgroups based on race and ethnicity exhibited varied depression prevalence rates (n=41, 38.32%). In the NHW subgroup (n=53), 22 (41.51%) had a history of depression, while 31 (58.49%) had no history. Among the NHB subgroup (n=27), 7 (25.93%) had a history of depression, while 20 (74.07%) had no history. In the Hispanic subgroup (n=26), 11 (42.31%) individuals had a history of depression, whereas 15 (57.69%) had no history. A single participant in the “Other” subgroup had a history of depression.

**Table 2.** Prevalence of depression among metabolic and bariatric surgery candidates by recruitment method and race and ethnicity in a prospective cohort study (2019‐2022).

Variable and group	Participants (N=380), n (%)		*P* value
	History of depression	No history of depression	
Overall prevalence	115 (30.26)	265 (69.74)	.03^a^
Social media group (n=107)	41 (38.32)	66 (61.68)	—^b^
NHW^c^ (n=53)	22 (41.51)	31 (58.49)	
NHB^d^ (n=27)	7 (25.93)	20 (74.07)	
Hispanic (n=26)	11 (42.31)	15 (57.69)	
Other (n=1)	1 (100)	0 (0)	
Nonsocial media group (n=273)	74 (27.11)	199 (72.89)	—
NHW (n=98)	26 (26.53)	72 (73.47)	
NHB (n=120)	28 (23.33)	92 (76.67)	
Hispanic (n=41)	15 (36.59)	26 (63.41)	
Other (n=14)	5 (35.71)	9 (64.29)	

a There was a significant difference between depression prevalence in the social media group versus nonsocial media group.

b Not applicable.

c NHW: non-Hispanic White.

d NHB: non-Hispanic Black.

### Recruitment Method by Race and Ethnicity

Table 3 presents the odds of recruitment via social media, adjusting for age, sex, and BMI, stratified by race and ethnicity. In the unadjusted model, representing the crude odds, Hispanic participants had odds of 1.17 (95% CI 0.24‐0.71, *P*=.60) for being recruited via social media, while NHB participants had odds of 0.42 (95% CI 0.65‐2.12, *P*=.001), with NHW participants serving as the reference group.

In the adjusted model, adjusted for age, sex, BMI, education, and financial status, Hispanic participants had odds of 1.22 (95% CI 0.63‐2.37, *P*=.55) for being recruited via social media, while NHB participants had odds of 0.40 (95% CI 0.22‐0.72, *P*=.002). These findings indicate that after adjusting for demographic variables, NHB participants demonstrated significantly lower odds of recruitment via social media compared to NHW participants.

**Table 3.** Odds of being recruited via social media by race and ethnicity among metabolic and bariatric surgery candidates, adjusting for age, sex, BMI, education, and financial status.

Variables	Odds ratio (95% CI)	*P* value
Race/ethnicity		
Model 1^a^ (crude odds ratio)		
Hispanic	1.17 (0.24‐0.71)	.60
NHB^b^	0.42 (0.65‐2.12)	.001^c^
NHW^d^	1.0 (ref)	—^e^
Model 2^f^ (adjusted odds ratio)		
Hispanic	1.22 (0.63‐2.37)	.55
NHB	0.40 (0.22‐0.72)	.002^c^
NHW	1.0 (ref)	—

a Model 1: crude (social media as primary outcome).

b NHB: non-Hispanic Black.

c Significant.

d NHW: non-Hispanic White.

e Not applicable.

f Model 2: adjusted for age, sex, BMI, education, and financial status (social media as primary outcome).

### History of Depression and Social Media Recruitment

Univariate logistic models showed that individuals with a history of depression were 67% more likely (OR 1.67, 95% CI 1.04‐2.68, *P*=.03) to be recruited via social media than those with no history of depression (Table 4). However, after adjustment for age, sex, race and ethnicity, BMI, education, and financial status, the association between depression history and recruitment method was no longer significant. This indicated that one or more of these covariates influenced the relationship between our outcome variables, recruitment method, and depression history, warranting further subanalysis.

**Table 4.** Odds of depression for social media recruits, adjusting for age among metabolic and bariatric surgery candidates, race and ethnicity, sex, BMI, education, and financial status.

Variables	Odds ratio (95% CI)	*P* value
Depression history		
Model 1^a^ (crude odds ratio)		
Depression	1.67 (1.04‐2.68)	.03^b^
No depression	1.0 (ref)	—^c^
Model 2^d^ (adjusted odds ratio)		
Depression	1.51 (0.88‐2.62)	.13
No depression	1.0 (ref)	—

a Model 1: crude (social media as primary outcome).

b Significant.

c Not applicable.

d Model 2: adjusted for age, race/ethnicity, sex, BMI, education, and financial status (social media as primary outcome).

### Age, Depression, and Recruitment Method

After adjusting for sex, race and ethnicity, BMI, education, and financial status, participants with a history of depression who were below the mean age were over twice as likely (OR 2.26, 95%CI, 1.03‐4.95) to be recruited via social media compared to those with no history of depression (Table 5). However, with the same adjustments, no significant association was found among participants above the mean sample age.

**Table 5.** Relationship between age and recruitment method on odds of depression among metabolic and bariatric surgery candidates, after adjusting for sex, race and ethnicity, BMI, education, and financial status.

Variables	Adjusted odds ratio (95% CI)	*P* value
Depression history		
Model 1^a^		
Depression	2.26 (1.03‐4.95)	.04^b^
No depression	1.0 (ref)	—^c^
Model 2^d^		
Depression	1.13 (0.47‐2.73)	.79
No depression	1.0 (ref)	—

a Model 1: age below mean, adjusted for sex, race/ethnicity, BMI, education, and financial status (social media as primary outcome).

b Significant.

c Not applicable.

d Model 2: age above mean, adjusted for sex, race/ethnicity, BMI, education, and financial status (social media as primary outcome).

## Discussion

### Principal Findings

This study aimed to investigate the relationship between social media recruitment, depression, race, and ethnicity among individuals seeking MBS. Our analysis of 380 MBS candidates showed that participants recruited via social media exhibited a 67% higher likelihood of depression history compared to nonsocial media recruits. Furthermore, participants with a history of depression below the mean sample age were over twice as likely to be recruited via social media. Our findings also revealed significant disparities in social media recruitment among racial and ethnic groups. Hispanic and NHW participants were more likely to be recruited through social media than NHB. Specifically, adjusted models demonstrated a 60% lower likelihood of social media recruitment among NHB participants compared to NHW participants. These findings underscore the significance of exploring the interplay between social media, mental health disorders, and demographic factors in this population.

Our first key finding reveals a significant association between social media recruitment and a history of depression, particularly among younger individuals below the sample mean age, who were twice as likely to report such a history. Extensive studies have explored the relationship between social media usage and depression, emphasizing the heightened risk of depression, anxiety, and psychological distress associated with addictive or problematic platform use [23]. A systematic examination of 11 studies focusing on young individuals corroborates this, revealing a modest yet statistically significant association between social media usage and symptoms of depression [24]. The observation that younger individuals with a history of depression are more likely to be recruited via social media underscores the growing concern about the impact of social media on mental health, particularly in younger populations.

Other contributing factors between social media and depression include sleep impairment and sedentary behavior [25]. These factors, combined with the other potentially negative effects of problematic social media use, defined as a “nonsubstance–related disorder by which detrimental effects occur as a result of preoccupation and compulsion to excessively engage in social media platforms despite negative consequences” [26], may contribute to the higher prevalence of depression observed in our sample of MBS candidates. Addressing these issues through increased physical activity could offer protective benefits, as regular exercise has been shown to reduce depressive symptoms, support healthy weight management, and improve sleep quality [25,27-32]. While both depression and obesity are multifaceted conditions influenced by various biological, psychological, and social determinants, promoting physical activity represents a practical starting point for improving overall mental and physical health.

Our second key finding was that NHB participants were 60% less likely to be recruited via social media. This finding is particularly concerning given that NHB individuals bear a disproportionate burden of obesity and its health-related consequences. According to the National Center for Health Statistics, NHB women represent the demographic group with the highest risk for overweight, obesity, and severe obesity, followed closely by NHB men [33]. However, this underrepresentation is not surprising, given the significant history of distrust due to unethical practices that have occurred when enrolling this population in previous scientific studies. Prior research has indicated that NHB individuals are less inclined to participate in scientific studies overall [34]. Our results corroborate this disparity and underscore the challenges of using social media for recruitment in scientific research.

The efficacy of social media as a tool for recruiting diverse samples remains a topic of debate [11]. The success of such methods often depends on a comprehensive understanding of the target audience’s social media habits [35]. Recent research has shown promising results, with one study demonstrating a substantial increase in racial and ethnic diversity among participants recruited through social media for a clinical trial [36]. Other studies have raised concerns about potential biases, noting an overrepresentation of younger NHW individuals with higher education and income levels [37]. Our findings align with these observations, as we also reported a higher likelihood of recruiting Hispanic and NHW individuals through social media compared to NHB individuals. This result emphasizes the need for proactive measures to address potential biases in social media recruitment.

Additionally, obesity has been consistently associated with an increased risk of depression [38]. However, the significance of social support, whether received in person or through web-based platforms, cannot be overstated in optimizing positive outcomes for patients undergoing MBS. Previous research has underscored the positive correlation between heightened social support and decreased depression, along with improved weight loss among both pre- and post-MBS groups [39]. In one study, Facebook was used as a platform to deliver weight management program materials, resulting in documented weight loss among participants [40]. Furthermore, a 2016 study examining MBS-related Facebook support groups identified various recurring themes in online discussions, ranging from eating recommendations and postsurgery changes to anxiety, depression, and body image concerns [41]. The study concluded that online groups are valuable for fostering support, sharing experiences, and discussing challenges for pre- and post-MBS patients.

Overall, social media as a tool for research recruitment has several advantages, including a broad reach, cost-effectiveness, and the potential to foster social support related to weight management, eating disorders, and mental health. However, researchers must approach social media recruitment cautiously or risk recruiting homogenous samples and should be aware of social media’s association with depression. Combining more traditional recruitment methods with online recruitment can help ensure diverse study samples and enhance the validity, generalizability, and equity of findings.

Future research should explore culturally sensitive recruitment methods that include social media and other innovative techniques while continuing to examine the relationship between mental health and social media usage. Improved recruitment methods should consist of partnerships with community organizations, tailored outreach campaigns, and evaluations of effectiveness. As our digital world continues to evolve, enhancing recruitment diversity is critical to reducing health inequities and advancing the quality and fairness of clinical and public health research.

### Limitations and Strengths

Despite providing valuable insights, this study has limitations. One limitation of our study is that the sample was drawn exclusively from a single geographic region, which may impact the generalizability of the findings to a broader population. However, it is crucial to highlight that our sample includes a diverse participant population. Furthermore, our sample consisted specifically of MBS candidates, and we examined social media recruitment among this group and not social media use itself. All results should be interpreted with these key factors and limitations in mind.

### Conclusion

In conclusion, our findings show that MBS candidates recruited through social media had a higher prevalence of depression, particularly among younger individuals, and were more likely to be NHW or Hispanic. Therefore, our findings underscore the significance of using various recruitment methods to prevent bias and optimize and ensure sample diversity in research.

## Abbreviations

aOR: adjusted odds ratio
HIPAA: Health Insurance Portability and Accountability Act
MBS: metabolic and bariatric surgery
MINI: Mini International Neuropsychiatric Interview
NHB: non-Hispanic Black
NHW: non-Hispanic White
OR: odds ratio
REDCap: Research Electronic Data Capture
